# (*E*)-2-[(2-Formyl­phen­oxy)meth­yl]-3-(4-methyl­phen­yl)prop-2-ene­nitrile

**DOI:** 10.1107/S1600536811051415

**Published:** 2011-12-07

**Authors:** N. Manikandan, S. Murugavel, D. Kannan, M. Bakthadoss

**Affiliations:** a Department of Physics, Bharathidasan Engineering College, Nattrampalli, Vellore 635 854, India; b Department of Physics, Thanthai Periyar Government Institute of Technology, Vellore 632 002, India; c Department of Organic Chemistry, University of Madras, Chennai 600 025, India

## Abstract

In the title compound, C_18_H_15_NO_2_, the dihedral angle between the two benzene rings is 74.8 (1)°. The carbonitrile chain is almost linear, the C—C—N angle being 176.2 (2)°. In the crystal, π–π inter­actions [centroid–centroid distance = 3.842 (1) Å] are observed.

## Related literature

For background to the synthetic procedure, see: Bakthadoss & Murugan (2010 ▶). For related structures, see: Swaminathan *et al.* (2011 ▶); Prasanna *et al.* (2011 ▶).
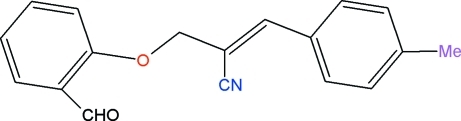


## Experimental

### 

#### Crystal data

C_18_H_15_NO_2_
*M* *_r_* = 277.31
Monoclinic, 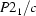

*a* = 7.0792 (4) Å
*b* = 13.7006 (7) Å
*c* = 15.3587 (9) Å
β = 96.782 (2)°
*V* = 1479.21 (14) Å^3^
*Z* = 4
Mo *K*α radiation
μ = 0.08 mm^−1^
*T* = 293 K
0.23 × 0.21 × 0.15 mm

#### Data collection

Bruker APEXII CCD diffractometer
Absorption correction: multi-scan (*SADABS*; Sheldrick, 1996 ▶) *T*_min_ = 0.982, *T*_max_ = 0.988
15027 measured reflections
3321 independent reflections
1950 reflections with *I* > 2σ(*I*)
*R*_int_ = 0.022

#### Refinement

*R*[*F* ^2^ > 2σ(*F* ^2^)] = 0.041
*wR*(*F* ^2^) = 0.133
*S* = 1.01
3321 reflections
191 parameters
H-atom parameters constrained
Δρ_max_ = 0.14 e Å^−3^
Δρ_min_ = −0.14 e Å^−3^

### 

Data collection: *APEX2* (Bruker, 2004 ▶); cell refinement: *APEX2* and *SAINT* (Bruker, 2004 ▶); data reduction: *SAINT* and *XPREP* (Bruker, 2004 ▶); program(s) used to solve structure: *SHELXS97* (Sheldrick, 2008 ▶); program(s) used to refine structure: *SHELXL97* (Sheldrick, 2008 ▶); molecular graphics: *ORTEP-3* (Farrugia (1997 ▶); software used to prepare material for publication: *SHELXL97* and *PLATON* (Spek, 2009 ▶).

## Supplementary Material

Crystal structure: contains datablock(s) global, I. DOI: 10.1107/S1600536811051415/im2342sup1.cif

Structure factors: contains datablock(s) I. DOI: 10.1107/S1600536811051415/im2342Isup2.hkl

Supplementary material file. DOI: 10.1107/S1600536811051415/im2342Isup3.cml

Additional supplementary materials:  crystallographic information; 3D view; checkCIF report

## Figures and Tables

**Fig. 1.**
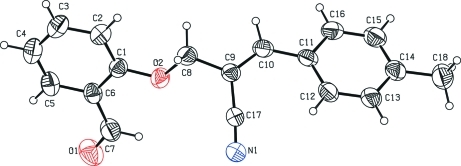
Molecular structure of the title compound showing displacement ellipsoids at the 30% probability level. H atoms are presented as a small spheres of arbitrary radii.

**Fig. 2.**
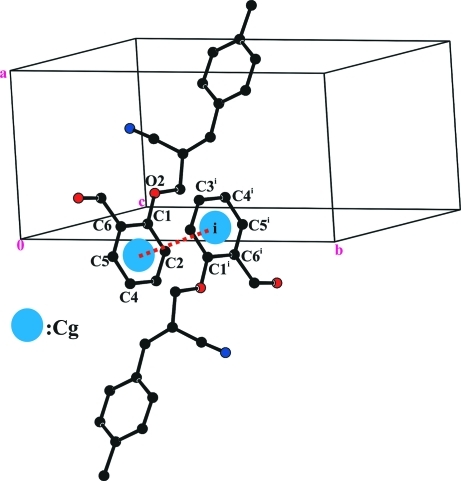
View of the π—π interactions (dotted lines) in the crystal structure of the title compound. Cg denotes the centroid of the C1–C6 benzene ring. [Symmetry code: (i) -x, 1-y, -z].

## supplementary crystallographic information

### Comment

The title compound is a stereodefined trisubstituted olefin, synthesized from the corresponding bromoderivative of a Baylis- Hillman adduct with salicylaldehyde *via* simple SN_2_ reaction in good yields. This *o*-salicyladehyde derivative is an important precursor for many heterocyclic frameworks (Bakthadoss *et al.*, 2010).

The title compound comprises a benzaldehyde moiety connected to a tolyl ring through a chain formed by a methoxy methyl and a propenenitrile group. The X-ray analysis confirms the molecular structure and atom connectivity as illustrated in Fig.1.

The dihedral angle between the two aromatic rings is 74.8 (1)°. The propenenitrile (N1/C17/C8–C11) plane forms dihedral angles of 53.6 (1)° and 22.7 (1)°, respectively, with the formyl phenyl and tolyl rings. The bond length C9—C17 [1.431 (2) Å] is significantly shorter than the expected value for a C—C single bond because of conjugation effects (Prasanna *et al.*, 2011). The carbonitrile side chain (C9–C17–N1) is almost linear, with the angle around the central carbon atom being 176.2 (2)°. The geometric parameters of the title molecule agree well with those reported for similar structures (Swaminathan *et al.*, 2011, Prasanna *et al.*, 2011).

The crystal packing (Fig. 2) is stabilized by intermolecular π—π interactions with a Cg—Cg^i^ seperation of 3.842 (1) Å [Fig. 2; Cg is the centroid of the C1–C6 benzene ring, symmetry code as in Fig. 2].

### Experimental

A solution of salicylaldehyde (1.0 mmol, 0.12 g) and potassium carbonate (1.5 mmol, 0.207 g) in acetonitrile was stirred for 15 minutes at room temperature. To this solution, (*E*)-2-(bromomethyl)-3-(4-methylphenyl)prop-2-enenitrile (1.2 mmol, 0.28 g) was added dropwise till the addition is complete. After the completion of the reaction, as indicated by TLC, acetonitrile was evaporated. EtOAc (15 ml) and water (15 ml) were added to the crude mass. The organic layer was dried over anhydrous sodium sulfate. Removal of solvent led to a crude product, which was purified through a pad of silica gel (100–200 mesh) using ethylacetate and hexanes (1:9) as solvents. The pure title compound was obtained as a colourless solid (0.24 g, 86 % yield). Recrystallization was carried out using ethylacetate as the solvent.

### Refinement

H atoms were positioned geometrically, with C–H = 0.93–0.97 Å and constrained to ride on their parent atom, with *U*_iso_(H) =1.5*U*_eq_ for methyl H atoms and 1.2*U*_eq_(C) for the other H atoms.

### Crystal data

**Table:**

C_18_H_15_NO_2_	*F*(000) = 584
*M**_r_* = 277.31	*D*_x_ = 1.245 Mg m^−^^3^
Monoclinic, *P*2_1_/*c*	Mo *K*α radiation, λ = 0.71073 Å
Hall symbol: -P 2ybc	Cell parameters from 3403 reflections
*a* = 7.0792 (4) Å	θ = 2.0–27.5°
*b* = 13.7006 (7) Å	µ = 0.08 mm^−^^1^
*c* = 15.3587 (9) Å	*T* = 293 K
β = 96.782 (2)°	Block, yellow
*V* = 1479.21 (14) Å^3^	0.23 × 0.21 × 0.15 mm
*Z* = 4	

### Data collection

**Table:**

Bruker APEXII CCD diffractometer	3321 independent reflections
Radiation source: fine-focus sealed tube	1950 reflections with *I* > 2σ(*I*)
graphite	*R*_int_ = 0.022
Detector resolution: 10.0 pixels mm^-1^	θ_max_ = 27.6°, θ_min_ = 2.0°
ω scans	*h* = −9→9
Absorption correction: multi-scan (*SADABS*; Sheldrick, 1996)	*k* = −17→17
*T*_min_ = 0.982, *T*_max_ = 0.988	*l* = −19→19
15027 measured reflections	

### Refinement

**Table:**

Refinement on *F*^2^	Primary atom site location: structure-invariant direct methods
Least-squares matrix: full	Secondary atom site location: difference Fourier map
*R*[*F*^2^ > 2σ(*F*^2^)] = 0.041	Hydrogen site location: inferred from neighbouring sites
*wR*(*F*^2^) = 0.133	H-atom parameters constrained
*S* = 1.01	*w* = 1/[σ^2^(*F*_o_^2^) + (0.0553*P*)^2^ + 0.2089*P*] where *P* = (*F*_o_^2^ + 2*F*_c_^2^)/3
3321 reflections	(Δ/σ)_max_ < 0.001
191 parameters	Δρ_max_ = 0.14 e Å^−^^3^
0 restraints	Δρ_min_ = −0.14 e Å^−^^3^

### Special details

**Table:**

Geometry. All esds (except the esd in the dihedral angle between two l.s. planes) are estimated using the full covariance matrix. The cell esds are taken into account individually in the estimation of esds in distances, angles and torsion angles; correlations between esds in cell parameters are only used when they are defined by crystal symmetry. An approximate (isotropic) treatment of cell esds is used for estimating esds involving l.s. planes.
Refinement. Refinement of F^2^ against ALL reflections. The weighted R-factor wR and goodness of fit S are based on F^2^, conventional R-factors R are based on F, with F set to zero for negative F^2^. The threshold expression of F^2^ > 2sigma(F^2^) is used only for calculating R-factors(gt) etc. and is not relevant to the choice of reflections for refinement. R-factors based on F^2^ are statistically about twice as large as those based on F, and R- factors based on ALL data will be even larger.

### Fractional atomic coordinates and isotropic or equivalent isotropic displacement parameters (Å^2^)

**Table:**

	*x*	*y*	*z*	*U*_iso_*/*U*_eq_	
N1	0.6118 (2)	0.26454 (11)	0.25898 (11)	0.0828 (5)	
O1	0.2658 (2)	0.23139 (12)	−0.09622 (11)	0.1133 (5)	
O2	0.25632 (15)	0.38860 (8)	0.11791 (7)	0.0726 (3)	
C1	0.0835 (2)	0.37959 (11)	0.06904 (11)	0.0621 (4)	
C2	−0.0823 (2)	0.42103 (13)	0.09048 (12)	0.0750 (5)	
H2	−0.0825	0.4565	0.1421	0.090*	
C3	−0.2478 (2)	0.40934 (15)	0.03456 (14)	0.0856 (6)	
H3	−0.3599	0.4372	0.0489	0.103*	
C4	−0.2505 (3)	0.35721 (16)	−0.04214 (14)	0.0879 (6)	
H4	−0.3631	0.3502	−0.0794	0.105*	
C5	−0.0866 (3)	0.31602 (14)	−0.06293 (12)	0.0777 (5)	
H5	−0.0879	0.2812	−0.1150	0.093*	
C6	0.0820 (2)	0.32522 (11)	−0.00780 (11)	0.0648 (4)	
C7	0.2538 (3)	0.27629 (15)	−0.02978 (14)	0.0846 (5)	
H7	0.3621	0.2802	0.0107	0.101*	
C8	0.2646 (2)	0.43560 (13)	0.20139 (11)	0.0716 (5)	
H8A	0.1879	0.4003	0.2392	0.086*	
H8B	0.2167	0.5018	0.1945	0.086*	
C9	0.4680 (2)	0.43625 (11)	0.24015 (10)	0.0627 (4)	
C10	0.5640 (2)	0.51893 (12)	0.25968 (10)	0.0691 (5)	
H10	0.4965	0.5755	0.2432	0.083*	
C11	0.7555 (2)	0.53562 (11)	0.30210 (10)	0.0645 (4)	
C12	0.8639 (3)	0.46728 (13)	0.35274 (12)	0.0802 (5)	
H12	0.8151	0.4051	0.3595	0.096*	
C13	1.0416 (3)	0.49015 (14)	0.39295 (13)	0.0867 (6)	
H13	1.1104	0.4430	0.4268	0.104*	
C14	1.1218 (3)	0.58109 (14)	0.38479 (12)	0.0775 (5)	
C15	1.0151 (3)	0.64806 (14)	0.33374 (14)	0.0858 (6)	
H15	1.0656	0.7097	0.3260	0.103*	
C16	0.8365 (3)	0.62670 (12)	0.29389 (13)	0.0799 (5)	
H16	0.7680	0.6744	0.2606	0.096*	
C17	0.5526 (2)	0.34163 (12)	0.25245 (11)	0.0626 (4)	
C18	1.3145 (3)	0.60602 (18)	0.43169 (16)	0.1045 (7)	
H18A	1.3777	0.6508	0.3967	0.157*	
H18B	1.3890	0.5476	0.4411	0.157*	
H18C	1.2998	0.6356	0.4872	0.157*	

### Atomic displacement parameters (Å^2^)

**Table:**

	*U*^11^	*U*^22^	*U*^33^	*U*^12^	*U*^13^	*U*^23^
N1	0.0778 (10)	0.0609 (9)	0.1058 (12)	−0.0005 (7)	−0.0062 (8)	−0.0046 (8)
O1	0.1137 (12)	0.1266 (12)	0.1033 (12)	−0.0130 (9)	0.0288 (9)	−0.0442 (10)
O2	0.0653 (7)	0.0851 (8)	0.0650 (7)	0.0095 (5)	−0.0017 (5)	−0.0154 (6)
C1	0.0592 (9)	0.0628 (9)	0.0630 (10)	−0.0010 (7)	0.0026 (8)	0.0060 (8)
C2	0.0696 (11)	0.0788 (11)	0.0761 (12)	0.0068 (8)	0.0068 (9)	0.0040 (9)
C3	0.0615 (11)	0.0977 (14)	0.0965 (15)	0.0056 (9)	0.0051 (10)	0.0173 (12)
C4	0.0681 (12)	0.1052 (15)	0.0864 (14)	−0.0147 (10)	−0.0076 (10)	0.0145 (12)
C5	0.0758 (12)	0.0859 (12)	0.0700 (12)	−0.0228 (9)	0.0022 (9)	0.0022 (9)
C6	0.0651 (10)	0.0634 (9)	0.0656 (10)	−0.0123 (7)	0.0067 (8)	0.0028 (8)
C7	0.0784 (12)	0.0901 (13)	0.0856 (14)	−0.0107 (10)	0.0112 (10)	−0.0195 (11)
C8	0.0733 (10)	0.0748 (11)	0.0655 (11)	0.0118 (8)	0.0038 (8)	−0.0101 (8)
C9	0.0720 (10)	0.0596 (9)	0.0554 (9)	0.0067 (7)	0.0026 (7)	−0.0061 (7)
C10	0.0839 (11)	0.0567 (9)	0.0656 (10)	0.0108 (8)	0.0037 (9)	−0.0036 (8)
C11	0.0798 (11)	0.0495 (8)	0.0640 (10)	0.0044 (7)	0.0078 (8)	−0.0086 (7)
C12	0.1049 (14)	0.0552 (9)	0.0741 (11)	−0.0040 (9)	−0.0157 (10)	−0.0031 (8)
C13	0.1041 (14)	0.0701 (11)	0.0791 (13)	0.0091 (10)	−0.0170 (11)	−0.0103 (9)
C14	0.0785 (11)	0.0775 (12)	0.0773 (12)	0.0015 (9)	0.0130 (9)	−0.0236 (10)
C15	0.0853 (13)	0.0634 (11)	0.1111 (16)	−0.0060 (9)	0.0212 (12)	−0.0088 (11)
C16	0.0834 (12)	0.0572 (10)	0.0991 (14)	0.0075 (8)	0.0105 (10)	0.0000 (9)
C17	0.0622 (9)	0.0593 (10)	0.0650 (10)	−0.0037 (7)	0.0013 (7)	−0.0061 (8)
C18	0.0862 (14)	0.1194 (17)	0.1068 (17)	−0.0062 (12)	0.0066 (12)	−0.0322 (14)

### Geometric parameters (Å, °)

**Table:**

N1—C17	1.1362 (19)	C9—C10	1.337 (2)
O1—C7	1.203 (2)	C9—C17	1.431 (2)
O2—C1	1.3634 (17)	C10—C11	1.451 (2)
O2—C8	1.4297 (19)	C10—H10	0.9300
C1—C2	1.378 (2)	C11—C16	1.385 (2)
C1—C6	1.395 (2)	C11—C12	1.389 (2)
C2—C3	1.378 (2)	C12—C13	1.371 (2)
C2—H2	0.9300	C12—H12	0.9300
C3—C4	1.376 (3)	C13—C14	1.381 (3)
C3—H3	0.9300	C13—H13	0.9300
C4—C5	1.362 (3)	C14—C15	1.374 (3)
C4—H4	0.9300	C14—C18	1.505 (3)
C5—C6	1.385 (2)	C15—C16	1.370 (3)
C5—H5	0.9300	C15—H15	0.9300
C6—C7	1.462 (3)	C16—H16	0.9300
C7—H7	0.9300	C18—H18A	0.9600
C8—C9	1.492 (2)	C18—H18B	0.9600
C8—H8A	0.9700	C18—H18C	0.9600
C8—H8B	0.9700		
			
C1—O2—C8	118.26 (12)	C17—C9—C8	114.60 (13)
O2—C1—C2	124.43 (15)	C9—C10—C11	131.12 (15)
O2—C1—C6	115.50 (14)	C9—C10—H10	114.4
C2—C1—C6	120.06 (15)	C11—C10—H10	114.4
C3—C2—C1	119.25 (18)	C16—C11—C12	116.81 (16)
C3—C2—H2	120.4	C16—C11—C10	118.41 (15)
C1—C2—H2	120.4	C12—C11—C10	124.76 (16)
C4—C3—C2	121.25 (18)	C13—C12—C11	121.01 (17)
C4—C3—H3	119.4	C13—C12—H12	119.5
C2—C3—H3	119.4	C11—C12—H12	119.5
C5—C4—C3	119.34 (17)	C12—C13—C14	122.04 (18)
C5—C4—H4	120.3	C12—C13—H13	119.0
C3—C4—H4	120.3	C14—C13—H13	119.0
C4—C5—C6	121.04 (18)	C15—C14—C13	116.75 (18)
C4—C5—H5	119.5	C15—C14—C18	121.87 (19)
C6—C5—H5	119.5	C13—C14—C18	121.36 (19)
C5—C6—C1	119.04 (16)	C16—C15—C14	121.92 (18)
C5—C6—C7	119.82 (17)	C16—C15—H15	119.0
C1—C6—C7	121.13 (15)	C14—C15—H15	119.0
O1—C7—C6	125.05 (18)	C15—C16—C11	121.46 (17)
O1—C7—H7	117.5	C15—C16—H16	119.3
C6—C7—H7	117.5	C11—C16—H16	119.3
O2—C8—C9	107.21 (13)	N1—C17—C9	176.18 (17)
O2—C8—H8A	110.3	C14—C18—H18A	109.5
C9—C8—H8A	110.3	C14—C18—H18B	109.5
O2—C8—H8B	110.3	H18A—C18—H18B	109.5
C9—C8—H8B	110.3	C14—C18—H18C	109.5
H8A—C8—H8B	108.5	H18A—C18—H18C	109.5
C10—C9—C17	122.98 (14)	H18B—C18—H18C	109.5
C10—C9—C8	122.40 (14)		
			
C8—O2—C1—C2	6.6 (2)	O2—C8—C9—C17	−59.52 (19)
C8—O2—C1—C6	−174.26 (14)	C17—C9—C10—C11	−6.1 (3)
O2—C1—C2—C3	178.05 (15)	C8—C9—C10—C11	175.59 (16)
C6—C1—C2—C3	−1.0 (2)	C9—C10—C11—C16	163.39 (19)
C1—C2—C3—C4	0.0 (3)	C9—C10—C11—C12	−18.2 (3)
C2—C3—C4—C5	0.3 (3)	C16—C11—C12—C13	0.6 (3)
C3—C4—C5—C6	0.5 (3)	C10—C11—C12—C13	−177.83 (17)
C4—C5—C6—C1	−1.5 (3)	C11—C12—C13—C14	−0.5 (3)
C4—C5—C6—C7	177.08 (18)	C12—C13—C14—C15	−0.4 (3)
O2—C1—C6—C5	−177.37 (13)	C12—C13—C14—C18	178.02 (19)
C2—C1—C6—C5	1.8 (2)	C13—C14—C15—C16	1.1 (3)
O2—C1—C6—C7	4.1 (2)	C18—C14—C15—C16	−177.31 (19)
C2—C1—C6—C7	−176.79 (16)	C14—C15—C16—C11	−1.0 (3)
C5—C6—C7—O1	4.5 (3)	C12—C11—C16—C15	0.1 (3)
C1—C6—C7—O1	−176.95 (19)	C10—C11—C16—C15	178.63 (17)
C1—O2—C8—C9	−179.60 (13)	C10—C9—C17—N1	−160 (3)
O2—C8—C9—C10	118.94 (17)	C8—C9—C17—N1	19 (3)
